# Zonation of Microbial Communities by a Hydrothermal Mound in the Atlantis II Deep (the Red Sea)

**DOI:** 10.1371/journal.pone.0140766

**Published:** 2015-10-20

**Authors:** Yong Wang, Jiang Tao Li, Li Sheng He, Bo Yang, Zhao Ming Gao, Hui Luo Cao, Zenon Batang, Abdulaziz Al-Suwailem, Pei-Yuan Qian

**Affiliations:** 1 Sanya Institute of Deep Sea Science and Engineering, Chinese Academy of Sciences, San Ya, China; 2 Division of Life Science, Hong Kong University of Science and Technology, Clear Water Bay, Hong Kong, China; 3 State Key Laboratory of Marine Geology, Tongji University, Shanghai, China; 4 Computational Biosciences Research Center, King Abdullah University of Science and Technology, Thuwal, Saudi Arabia; Wageningen University, NETHERLANDS

## Abstract

In deep-sea geothermal rift zones, the dispersal of hydrothermal fluids of moderately-high temperatures typically forms subseafloor mounds. Major mineral components of the crust covering the mound are barite and metal sulfides. As a result of the continental rifting along the Red Sea, metalliferous sediments accumulate on the seafloor of the Atlantis II Deep. In the present study, a barite crust was identified in a sediment core from the Atlantis II Deep, indicating the formation of a hydrothermal mound at the sampling site. Here, we examined how such a dense barite crust could affect the local environment and the distribution of microbial inhabitants. Our results demonstrate distinctive features of mineral components and microbial communities in the sediment layers separated by the barite crust. Within the mound, archaea accounted for 65% of the community. In contrast, the sediments above the barite boundary were overwhelmed by bacteria. The composition of microbial communities under the mound was similar to that in the sediments of the nearby Discovery Deep and marine cold seeps. This work reveals the zonation of microbial communities after the formation of the hydrothermal mound in the subsurface sediments of the rift basin.

## Introduction

**Fig 1:**
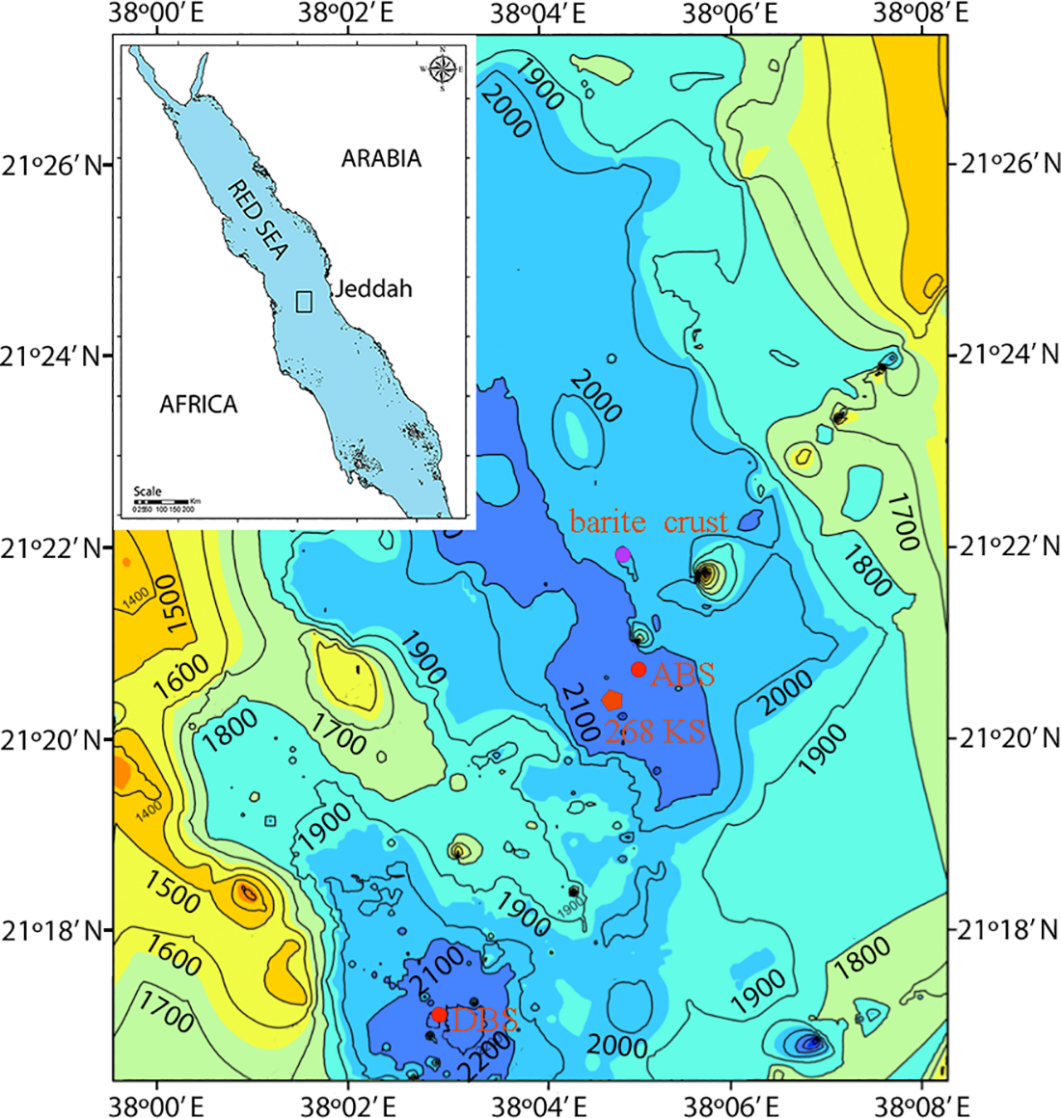
Geographic locations of two deep sea brine pools in the Red Sea. The sediment cores from Atlantis II (ABS) and Discovery (DBS) were sectioned for microbial studies. A barite crust was identified between 152 cm and 169 cm by mineral analysis on the ABS. The core of 268 KS was sampled in 1982 by Thisse et al. [19]. The barite crust in the Atlantis II Deep was discovered by Sval'nov et al. (1984) [10].

Across the seafloor of the Red Sea, there are a total of 25 brine pools [1,2]. The Atlantis II brine pool was discovered at a depth of approximately 2100 m and had been extensively studied since the 1960s [1,3–5]. Metal sulfides have accumulated in approximately 170 meters of anoxic brine water. The pool of preserved metal sulfides has attracted attention because of its mineral value. However, hydrothermal vents on the floor of the pool have yet to be found [6]. It was suggested that hydrothermal solutions penetrate the thick covering of the lithosphere and sediments from the continental margin before seeping out. The thermal sources in such geographical conditions were believed to be released through narrow tectonic faults and sediments, which ultimately resulted in hydrothermal mounds [7]. A barite crust formed after the eruption of hydrothermal solutions and served as an upper boundary of the mound [8]. The barite crust was capped with anhydrite cones, and metalliferous deposits subsequently accumulated. Under the barite, massive sulfide deposits were separated to some extent from the overlying sediments [7]. In the southwestern portion of the Atlantis II pool, a barite crust was discovered in 1984 and mineral and chemical features of this layer, together with the isotope composition of sulfur and oxygen, support their hydrothermal origin [9] (Fig 1). This barite crust was produced close to an orifice of an intermittent hydrothermal vent as a result of chemogenic precipitation. The accumulated metal sulfides in the brine sediment of the Atlantis II could not be easily oxidized due to the coverage by a large body of anoxic brine water.

The sediment within the mound was relatively isolated from the overlying part, although hydrothermal solutions may erupt to the upper layer through vein networks. As a result, formation of the hydrothermal mound may greatly influence the inhabitants of the sediments due to its capacity to alter the local environmental settings. It is then hypothesized that the formation of the barite crust may increase the segregation of microbial communities in the hydrothermal sediments. Above the barite crust, hydrothermal components are not major chemical sources that support chemolithotrophs. As metal sulfides accumulate on the mound, the biological communities probably change gradually. Under the mound, the original inhabitants will further evolve in the hotter and hypersaline environments in a long period of time. At present, such a hypothetical effect of the hydrothermal mound on the shift in microbial communities has never been examined. In the Red Sea, the distribution and structure of the hydrothermal mounds have not been elucidated. Thus, inhabitants under the mound remain an unknown biological world.

In 2010, we sampled a sediment core in the southwestern zone of the Atlantis II Deep. Mineral analysis revealed a barite crust, indicating the presence of a hydrothermal mound. In this study, we present mineral evidence for the presence of a hydrothermal mound in the Atlantis II Deep and reveal the stratifying effect of the mound on the microbial communities.

## Materials and Methods

### Sampling

**Fig 2:**
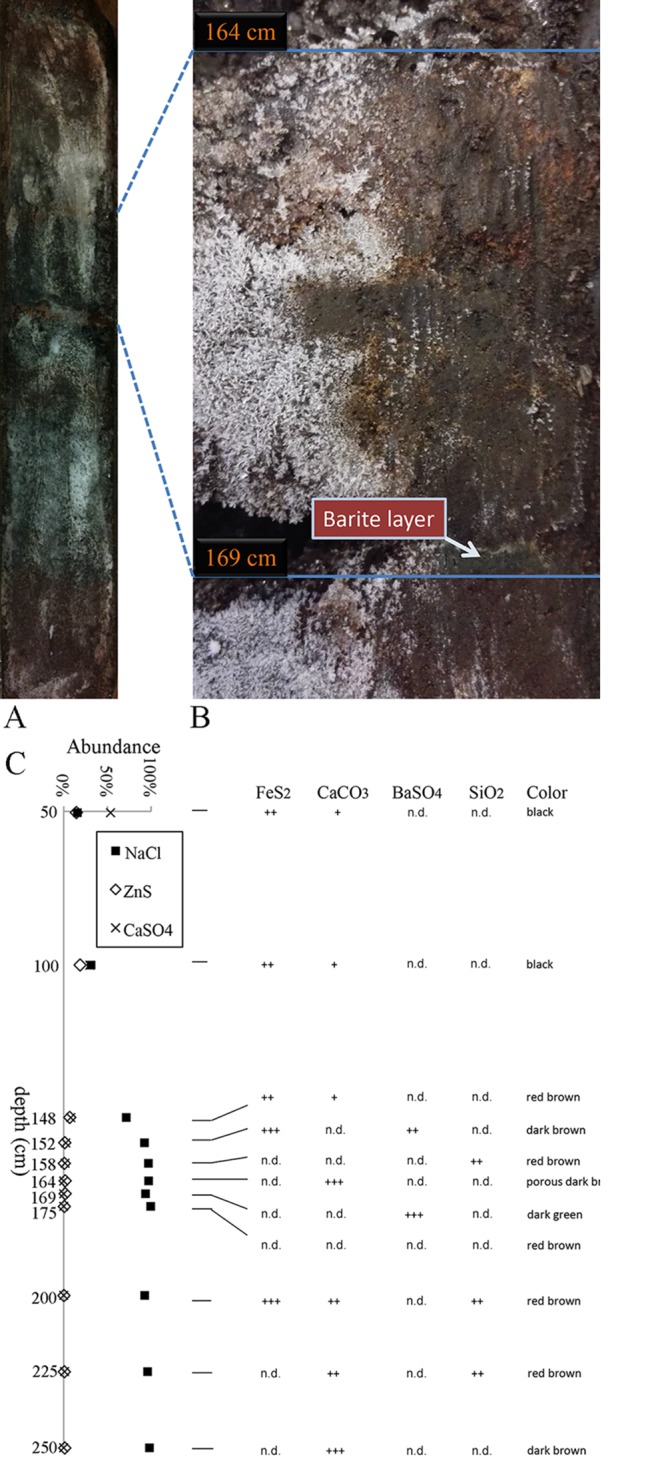
Mineral components of a sediment core from the Atlantis II Deep of the Red Sea. The core (A) was obtained from the Atlantis II Deep (see Fig 1). The layers of interests were selected based on naked eye observation (B). XRD analysis shows that the major components were anhydrite, halite and sphalerite (C). The abundance and distribution of the other minor components FeS_2_, CaCO_3_, BaSO_4_ and SiO_2_ were indicated by + (<10%), ++ (10%-30%) and +++ (>30%), with halite content discounted (C). Minor components that could not be detected were labeled as 'n.d.'.

No specific permissions were required for these locations/activities. This field studies did not involve endangered or protected species in the two sampling sites. During the R/V *Aegaeo* cruise leg III in April 2010, gravity sediment cores were obtained from the Atlantis II Deep (21°20.73’ N, 38°05.04’ E) and the Discovery Deep (21°17.09’ N, 38°02.90’ E) in the Red Sea (Fig 1). The core from the Atlantis II was approximately 2.5 meters (Fig 2A), and the one from the Discovery was 3.5 meters in length. They were sectioned into small portions of 50 cm. Several layers were selected based on the colors and physical characteristics observed with the naked eye and were then subjected to further mineral, chemical and microbiological studies.

### Mineral and chemical analyses

Five grams of the sediment samples from the Atlantis II were dried at 50°C and thoroughly ground to a fine powder (~0.074 mm) using a mortar and a pestle. X-ray diffraction (XRD) pattern analysis was then performed using a Rint 2000 X-ray diffractometer (using Cu Kα radiation at 40 kV and 30 mA, scanning from 2° to 80° with a velocity of 1° 2θ/min). Diffraction angles (referred to as ′2θ′) corresponding to the atomic structure unique to each mineral were measured. The compositions of the mineral components were converted to wt% in each layer of interest. The concentrations of dissolved organic and inorganic carbons (DOC and IC) in the pore water were determined using a TOC-V analyzer (Shimadzu, Kyoto, Japan). The sulfate, nitrite and nitrate concentrations were measured using ion chromatography (Shimadzu, Kyoto, Japan).

### Pyrosequencing and analysis of 16S rRNA sequence amplicons

Ten grams of the sediments were used for DNA extraction and purification using a MOBIO PowerMaxSoil DNA Isolation Kit following the manufacturer’s instruction. The DNA sample was quantified with a PicoGreen dsDNA Quantitation Kit (Life Technologies, Carlsbad, CA, USA). The total extracted DNA was used as the template for PCR amplification of 16S rRNA genes using 8-nt barcoded primers designed with Barcrawl [10]. A set of universal primers was applied for the amplification: forward primer U905F (5′- TGAAACTYAAAGGAATTG-3′) and reverse primer R1492 (5′- GGTTACCTTGTTACGACTT-3′)[11]. The primers target hypervariable regions V6 to V9 of the 16S rRNA genes (rDNA). The PCR reactions were conducted in a thermocycler (Bio-Rad, Hercules, CA, USA) under the following conditions: initial denaturation at 94°C for 5 min; 25 cycles of 94°C for 50 s, 53°C for 50 s, and 72°C for 50 s; and a final extension at 72°C for 6 min. The PCR products were purified with an agarose gel purification kit (TaKaRa, Dalian, China) and quantified using a NanoDrop ND-1000 device (Nanodrop, DE, USA). Two PCR replicates were performed for each sample. The mixture of 16S rDNA amplicons of 42 samples from Atlantis II, Discovery and other sediment samples was used for 454 FLX Titanium pyrosequencing (Roche, Basel, Switzerland). The pyrosequencing data were deposited in the NCBI SRA database under accession number SRP040612 (run number SRR1491769).

The downstream analysis of the pyrosequenced amplicon reads was performed using QIIME 1.7.0 [12]. Reads of low quality were filtered out by enforcing the following quality control criteria: 1) Exclusion of reads with one or more ambiguous nucleotides; 2) Exclusion of reads shorter than 150 bp; 3) Exclusion of reads containing homopolymers of 6 bp and above; 4) Exclusion of reads with an average flowgram score of 25 in a quality window of 50 bp. The qualified reads in average length of about 450 bp were *de novo* aligned with MUSCLE [13], followed by sorting into operational taxonomic units (OTUs) at a 3% dissimilarity. Chimeric reads were identified and excluded using ChimeraSlayer in the QIIME package after alignment with PyNAST [14] against the SILVA16S rRNA gene database (version 111) [15]. Chao1 and Shannon index for species richness and biodiversity estimates were calculated at a 3% dissimilarity following the instructions of the QIIME package. Weighted UniFrac metrics were used for the principal coordinate analysis (PCoA). Taxonomy assignment was determined using the RDP classifier against the SILVA 16S rRNA gene database with a confidence of 80%. To accurately determine the phylogenetic status of the two most dominant archaeal groups, five representative sequences (accession numbers: KM102752-KM102756) of the OTUs (assigned at a 3% dissimilarity level) from the two archaeal groups were aligned with reference sequences from GenBank. The length of the representative sequences ranged between 559 and 573 bp, which was the minimum length used for selection of the reference sequences derived from the closest relatives in the NCBI. Maximum-likelihood (ML) phylogenetic trees with bootstrap values based on 500 replicates were constructed using the MEGA 5.1 program using GTR model [16]. A consensus neighbor-joining tree based on 1000 replicates served as the reference tree for the ML method.

## Results

### Discovery of a barite crust in the sediment of the Atlantis II Deep

The upper section (0–148 cm) of the sediment core from the Atlantis II Deep was uniformly black. The lower section (>148 cm) was red brown and was interrupted by dark brown and dark green layers (Fig 2A). The boundary of the two sections occurred at 148–169 cm, where vast differences could be observed over a short distance. In particular, a dense green layer was noticed in the layer spanning 168–169 cm and was intersected by a thin white layer (Fig 2B).

The sediments at depths of 50 cm (ABS50) and 100 cm (ABS100) were composed mostly of halite (NaCl), sphalerite (ZnS), and anhydrite (CaSO_4_) (Fig 2C). Compared to ABS100, however, ABS50 contained higher anhydrite content and lower halite content. In the two layers, FeS_2_ was also present, but ferrous oxides were not detectable. Although the same components as in ABS50 and ABS100 were present in the 148-cm layer (ABS148), the low amount of Fe_2_O_3_ (4.9% of the total, roughly wt% hereafter) caused ABS148 to appear red brown. Approximately 72%of ABS148 was made up of halite, while halite content sharply increased to >95% at depth of 152 cm (ABS152). Thus, one of the important characteristics of the layers between 148 cm and 152 cm was a rapid increase of halite (Fig 2C). Between 152 cm and 169 cm (ABS169), nontronite, which caused the section to appear green, was detected, although its accurate proportion could not be determined (Fig 2A). In ABS152 and ABS169, barite comprised 22% and 72% of the layers, respectively, if halite was not considered. In the layer of 164 cm depth (ABS164), the porous sediment was mainly composed of carbonate (39%) and zinc sulfide (61%), again without considering halite. However, dissolved inorganic carbons (IC) concentration was not highest in ABS164. Instead, a strikingly high IC content at 12.6 mg/L in the pore water was shown in the layer at 158 cm (ABS158) (Table 1), several centimeters above the ABS164. In the sediment layers between ABS158-ABS169, dissolved organic carbons (DOC) concentration of the pore water declined dramatically from 33.9 mg/L to 14.9 mg/L (Table 1). Among all the layers, ABS169 was characterized with the lowest DOC concentration in the pore water. Quartz in a deep-sea sediment was also a typical geothermal product that was frequently detected under barite crust in hydrothermal mound [17,18]. Quartz appeared in ABS158, ABS200 and ABS225 (Fig 2C). All together, a barite crust was located between ABS152 and ABS169, and there was probably a hydrothermal mound approximating the sampling site.

**Table 1:** Porosity and carbon sources in the ABS sediment layers.

Depth (cm)	DOC (mg/L)	IC (mg/L)	SO_4_^2-^(mg/L)
**50**	32.8	6.4	20.0
**100**	31.0	6.3	13.7
**148**	39.1	3.3	15.2
**152**	27.4	6.6	16.6
**158**	33.9	12.6	17.8
**164**	18.0	2.3	10.7
**169**	14.9	3.2	12.8
**175**	15.9	2.6	8.78
**200**	21.4	2.3	8.82
**225**	40.1	6.3	12.7
**250**	46.5	5.4	13.18

The sediment layers were obtained from the Atlantis II Deep (ABS). DOC represents dissolved organic carbon; IC refers to inorganic carbon. Nitrite and nitrate were undetectable.

The 175-cm layer (ABS175) contained the largest amount of halite (99.5%), and the remaining components consisted mainly of ZnS. The predominance of halite persisted in the layers between ABS175 and ABS250 (Fig 2C). Ferrous oxides were mixed with FeS_2_ in the layers at 200 cm (ABS200) and 225 cm (ABS225), causing the two layers to be red brown. In all of the layers below ABS148, white anhydrite (gypsum sometimes) particles of different sizes up to 2 cm in diameter were frequently observed. The morphology and composition of the particles are similar to the description of anhydrites in core 268 KS that was sampled at a nearby site in 1982 [19] (Fig 1). With respect to mineral examinations on core 268 KS from the Atlantis II Deep, the anhydrite was postulated to be a result of a geyser-type discharge of hydrothermal fluids in the Deep [6]. Considering the short distance between the two sampling sites, the formation of anhydrites in ABS core was believed to be under similar geothermal conditions.

### Discontinuity of microbial communities at boundary of barite crust

**Fig 3:**
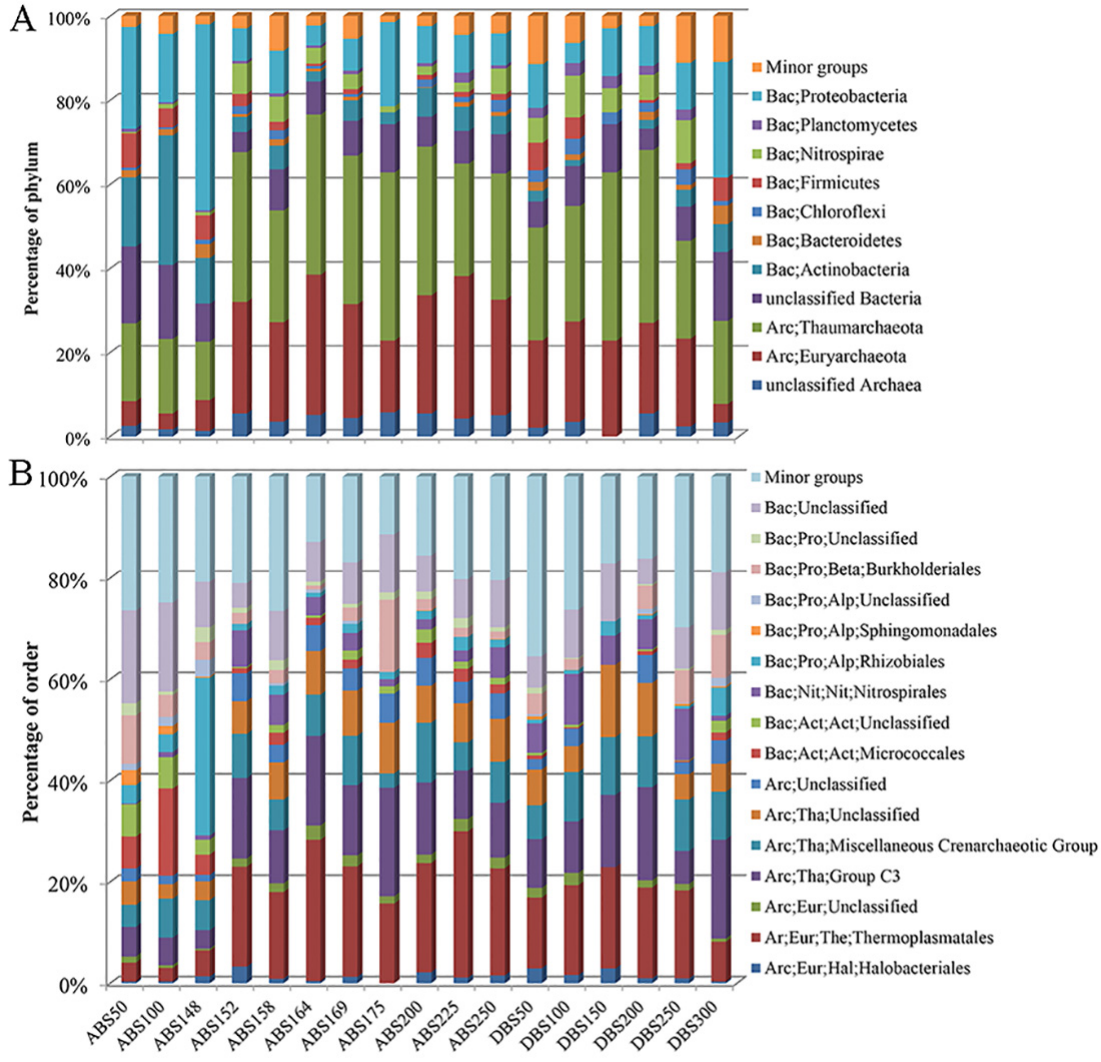
Microbial communities in two sediment cores from the central Red Sea. Minor groups were referred to the phyla accounted for<2% of all the communities. The compositions of the communities at phylum (A) and order (B) levels were based on sorting of the pyrosequenced 16S rRNA amplicons in SILVA database using confidence level of 80%. The sample IDs were referred to Table 2.

Microbial communities of the different sediment layers were revealed by pyrosequencing of 16S rDNA amplicons (Table 2). Comparison of the microbial communities at the phylum level between the layers showed a clear difference in composition of the communities between ABS148 and ABS152, as highlighted by the profound increase of archaeal relative abundance in the ABS152 layer and those below (Fig 3A). The average sequence relative abundance of *Archaea* in ABS50, ABS100 and ABS148 was 24.3% (standard deviation, 2.3%), whereas that of all the other ABS layers was 65%. In the top three layers, the low relative abundance of *Archaea* was attributable to the increase in *Proteobacteria*, *Actinobacteria*, and unclassified bacterial groups. Although *Bacteria*, as a whole, dominated these layers, the relative abundance of the bacterial phylum *Nitrospirae* declined in ABS50, ABS100, and ABS148, compared with the other ABS layers. For the ABS152 and other bottom layers, the relative abundance of *Halobacteriales* was markedly higher than that in the upper layers, which corresponds to the high halite component in these layers. The *Archaea* in these bottom layers were further assigned into orders and other lower levels, which resulted in the predominance of *Thermoplasmatales* and Thaumarchaeotic group C3 (Fig 3B).

**Table 2:** Pyrosequencing results of 16S rDNA amplicons.

Sample ID	No. reads	Barcode	No. OTUs	Chao1	Shannon index
**ABS50**	904	CGAGTCTC	336	235	5.5
**ABS100**	1427	ATGACGAC	444	235	5.3
**ABS148**	879	TCGTATGC	313	417	5.3
**ABS152**	317	TGCTCTCA	162	224	5.6
**ABS158**	1596	ATATGCGC	496	376	5.8
**ABS164**	509	CTCATCGA	258	252	5.7
**ABS169**	3834	GCTAGCTA	1125	325	5.8
**ABS175**	93	TCAGTGCA	45	124	5.1
**ABS200**	643	TGCAGAGC	333	304	5.7
**ABS225**	869	TCTGCATC	432	366	5.8
**ABS250**	3467	GCACACAC	1286	467	5.9
**DBS50**	594	TGCTCTCA	322	513	5.8
**DBS100**	2255	CTCATCGA	795	198	5.8
**DBS150**	36	TCAGTGCA	31	-	-
**DBS200**	655	GCTAGCTA	361	369	5.7
**DBS250**	655	ATATGCGC	286	378	5.8
**DBS300**	363	TGCAGAGC	184	366	5.7

The sediment samples were obtained from the Atlantis II Deep (ABS) and Discovery Deep (DBS) in 2010 cruise. The depths of the samples were inferred from the number (cm) in the sample IDs. Effective numbers of the 16S rDNA pyrosequencing reads were determined with barcodes on them. The number of OTUs was calculated based on a 3% dissimilarity among the aligned reads.

We also studied the microbial communities of the sediments from a neighboring brine pool, Discovery Deep (Table 2). Five layers ranging from 50 cm to 300 cm in depth also harbored high relative abundance of *Archaea*; bacteria represented a minority in these layers (Fig 3A). A considerable fraction of the *Archaea* could not be classified (Fig 3B). These results were consistent with the microbial composition of the Atlantis II sediment layers below 152 cm. However, the Discovery layers showed its specificities. Minor groups (<2% in all of the samples) occupied a larger fraction (about 11%) in deep layers of 250 cm (DBS250) and 300 cm (DBS300) than in the upper layers, indicating high species richness in the two bottom layers. The relative abundance of *Archaea* further decreased to <30% in DBS300, which results in a similar community of DBS300 to those in ABS50, ABS100, and ABS148 at the phylum level (Fig 3A). However, this was largely ascribed to the increase of *Proteobacteria* in these samples. At lower taxonomic levels, the taxa of *Proteobacteria* in DBS300 differed markedly from those in the three ABS layers and mainly affiliated with uncultured *Burkholderiaceae* and *Methylobacterium*, while *Ochrobactrum* was the most abundant genus of *Proteobacteria* in the three ABS samples.

The difference between the microbial communities was illustrated by PCoA analysis at OTUs level. PC1 and PC2 accounted for 70% of the sample variation. The three upper layers from ABS were separated from the other layers in the plot of PC1 and PC2 (S1 Fig).

### Dominance of archaea in the sample from a hydrothermal mound

**Fig 4:**
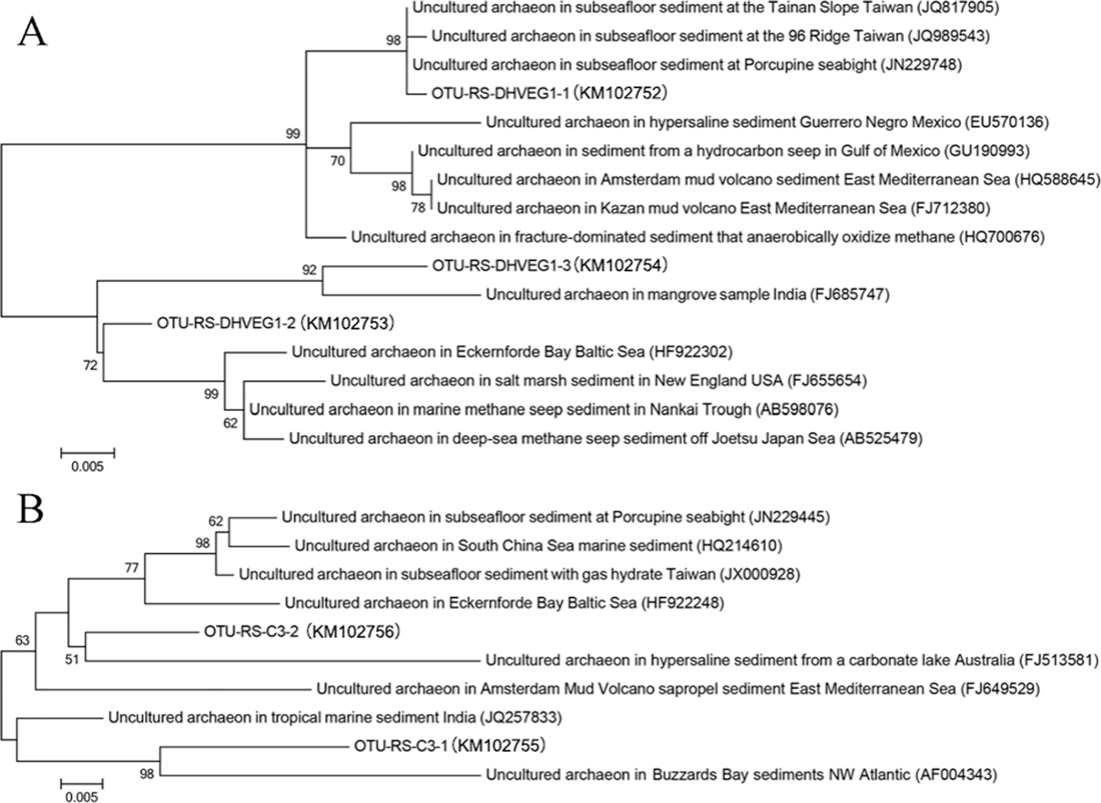
Phylogenetic trees of 16S rRNA gene sequences of archaeal groups DHVEG1 (A) and Thaumarchaeota group C3 (B) with their closest relatives. Three representative reads of the most abundant OTUs for deep-sea hydrothermal vent euryarchaeotic group 1 (OTU-RS-DHVEG1) and two for Thaumarchaeota group C3 (OTU-RS-C3) were used to identify their closest relatives in the GenBank. The phylogenetic trees were constructed using Maximum-Likelihood method.

As demonstrated above, the communities in the barite layers and in the mound resembled those of the sediments in the neighboring Discovery Deep. Phylogenetic analysis indicated that the two most abundant archaeal groups within the mound were deep-sea hydrothermal vent euryarchaeotic group 1 (DHVEG-1) and group C3 of the *Thaumarchaeota* [20,21]. They were also abundant in DBS but not in the upper layers of the ABS (S2 Fig). The phylogenetic relationships of the closest relatives in GenBank database indicated that the two archaeal groups were widespread in subsea floor sediment, particularly approximating methane seeps and mud volcanoes (Fig 4). The OTU-RS-DHVEG1-3 was the most abundant archaeal lineage in the two sediment cores and was relatively independent of the other sequences in the tree, suggesting the presence of novel lineages of DHVEG1in the two Deeps of the Red Sea.

Methanotrophs were frequently identified in the deep-sea anoxic sediments. The typical anaerobic methane consumers (ANME) [22,23] were examined in the different sediment communities. ANME-1 archaea were identified herein, whereas they were confined in the mound and the barite crust of ABS. They comprised between 0.1% and 0.9% in the present communities. The highest abundance was exhibited in a barite layer ABS164. On the other hand, the ANME-1 occupied approximately 0.2% of the corresponding communities in only two layers of the DBS.

## Discussion

In this study, the mineral components in the aforementioned layers indicate that the gravity core penetrated a hydrothermal mound in the Atlantis II Deep. The bottom of the barite crust was pinpointed at 169 cm of the sediment core; the upper boundary was at approximately 150 cm. The sediment core of 268 KS (Fig 1), which was studied by Ramboz et al. (1998) [6], was likely far away from the hydrothermal orifice, such that it did not contain the barite crust. The presence of the barite crust shown in this study likely resulted in the high halite content in the mound due to the over-saturation of salts under a high temperature (>68°C) in the Atlantis II Deep. A question is why there was nontronite in the ABS, an indication of the presence of a hydrothermal mound. In general, the hydrothermal mounds were formed by precipitation of Fe, Mn, and Si from hydrothermal fluids, which originated from the basalt crust and overlying sediments [7,17]. The Fe and Si precipitated as nontronite within the mounds under slightly reducing conditions. A deposition environment that produces nontronite (slow percolation of hydrothermal fluids through pelagic sediments) has been detected in the Galapagos Rift, in the Gulf of Aden and on the Mid-Atlantic Ridge [24]. Nontronites were also observed in two seamounts in the eastern Pacific where the hydrothermal fluids at low temperatures reacted directly with sea water [25]. Therefore, the deposition of nontronite in the ABS suggests the mixing of hydrothermal fluids with brine water at a moderate temperature (68°C) in the lower convective layer of the Atlantis II brine pool. In deep-sea environments, anhydrite facies generally form around hydrothermal vents [26]. A model has indicated that mild ongoing hydrothermal activities, for instance in the ABS, lead to a slow influx of solutions, followed by the formation of crystalline anhydrites in veins and around warm vents [26].

In this study, the discovery of quartz and ferrous oxides (Fe_2_O_3_ and Fe_3_O_4_) in the lower layers of the ABS provides additional evidence for the formation of the hydrothermal mound, because quartz was typically produced at a high temperature in all the hydrothermal mounds during volcanic activities [7,17]. Ferrous ions were likely oxidized in the mound under hydrothermal effects with electron acceptors. However, we could not detect nitrogen oxides (nitrate and nitrite) in these layers in this study. Although sulfate at low concentrations was detected in the sediment layers in the present study (Table 1), the source of sulfate in the anoxic sediment was questionable. The high concentration of metal ions in the sediment would have precipitated the sulfur oxides in the Atlantis II Deep [27].

In the present study, the subseafloor sediments in the mound were dominated by archaeal groups in the Atlantis II Deep. Several previous studies have demonstrated that archaea were overall at relatively low abundance in the microbial communities of the Atlantis II brine pool and sediment [28–30]. Explanations for this phenomenon have also been proposed in a previous report and will be further discussed below [29]. As previously reported, the concentration of hydrogen sulfide was extremely low in the brine pool [31], probably because the high ferrous concentration in the pool leads to the rapid depletion of hydrogen sulfide and subsequent precipitation of ferrous sulfide. Carbon dioxide may have been precipitated when it was first mixed with brine water that is rich in metal ions [27,29]. Thus, it has been concluded that autotrophic microbes are almost extinct in this metalliferous environment [29]. Hence, aromatic compounds in the sediment above the barite crust are probably the major carbon source, which caused the spreading of heterotrophs represented by *Ochrobactrum* sp. in the sediment of the Atlantis II Deep. *Ochrobactrum* species were able to anaerobically degrade polycyclic aromatic compounds [32,33]. Such a chemolithoheterotrophic lifestyle was in agreement with the *in situ* environment of the sediments of the Atlantis II Deep. As reported previously, aromatic compounds are accumulating in the Atlantis II brine pool as a result of hydrothermal maturation [34]. However, the archaeal communities detected by pyrosequencing of tagged 16S rDNA amplicons in this study were likely derived from free extracellular DNA preserved in the saline sediments. Previous studies have demonstrated that extracellular DNA could be well preserved in deep-sea sediments [35,36]. In this study, we did not conduct cell counting and fluorescence staining, and thus whether the DNA was extracted from living cells or trapped extracellular DNA may not be determined at present. The marine sediments containing fresh organic debris were usually associated with a high DOC in the pore water. In the present study, DOC was consistently low in the different studied layers, indicating that the microbes in ABS were living inhabitants.

The present study demonstrate that some of the archaeal inhabitants within and below the barite crust were also present in other marine cold-seep sediments [37], indicating that a sediment habitat with an environment to some extent similar to some cold seeps was maintained in the Atlantis II sediment. As the environment above the barite crust differed gradually from that in the mound, zonation of microbial communities occurred. Whether the archaea in the hydrothermal mound were the original microbial inhabitants after the formation of the mound remains to be answered.

At present, there are variant compositions of microbial communities in brine water and sediment layers in the Atlantis II Deep, in terms of dominant species and distribution patterns. There had been several studies on the microbial communities, but the results were variable. In a previous study, Siam et al. (2012) also identified archaeal groups in high relative abundance at the bottom of a sediment core from the Atlantis II Deep [30], which is another case of dominancy of *Archaea* in the Deep. Their results showed that the dominant archaeal inhabitants in the bottom layer (3.5 m to the seafloor) included Marine Benthic Group E, and ANME-1. The presence of the latter was confirmed by this study, but the former was not detected. In our previous metagenomic study on the Atlantis II sediment, *Cupriavidus* (*Betaproteobacteria*) and *Acinetobacter* (*Gammaproteobacteria*) were the most abundant species in the surface layer (12 cm) and bottom layer (222 cm) of a sediment core obtained in 2008 [38]. Both bacterial species were not the dominant inhabitants in the ABS core analyzed in the present study. Due to tremendous differences between brine water and sediment in the Deep, their microbial communities differ remarkably [28]. The lower convective layers of the Atlantis II and Discovery brine pools are dominated by *Gammaproteobacteria* [28], while *Alphaproteobacteria* and *Betaproteobacteria* are the major bacterial groups in the upper layers of Atlantis II sediment as shown in this study. All the above discrepancies in composition of microbial communities in the two Deeps were probably caused by 1) primer selection for amplification of rRNA genes; 2) different microenvironments in the sampling sites; 3) taxonomic assignment criteria employed by different studies; 4) different experimental procedures; and 5) sampling bias due to low biomass in sampling sites. Except for these potential problems, this study clearly demonstrates the profound changes in microbial communities in a deep-sea hydrothermal sediment under extensive mineralization process. Compared with other hydrothermal sediments, the Atlantis II hydrothermal field is unique in that sulfur and nitrogen oxides are low in the pore water of the sediments. This probably leads to lack of ANME that widely spread in other hydrothermal sediments as exemplified by the Guaymas Basin [39]. Also major proteobacterial groups differed between Atlantis II Deep and Guaymas Basin. Taxonomic assignment of 16S rRNA cloning sequences indicated that Guaymas Basin was occupied by *Gammaproteobacteria*, *Deltaproteobacteria* and *Epsilonproteobacteria*. Therefore, different geochemical conditions of hydrothermal sediments across the deepsea subseafloor resulted in various niche-specific microbial communities.

## Supporting Information

S1 Fig Principal component analysis (PCoA) of microbial communities. (DOCX)

S2 Fig Relative abundance of DHVEG1 and group C3. The relative abundance of the DHVEG1 and Thaumarchaeota group C3 was shown as their percentage in the individual communities. The sample IDs were referred to Table 2.
(DOCX)
