# Supplementary material for: Zonation of Microbial Communities by a Hydrothermal Mound in the Atlantis II Deep (the Red Sea)
Source: PLoS One. 2015 Oct 20;10(10):e0140766. doi: 10.1371/journal.pone.0140766 (PMC4613831; doi:10.1371/journal.pone.0140766)
Supplement: S1 Fig — (DOCX) [file pone.0140766.s001.docx]

S1 Fig. Principal component analysis (PCoA) of microbial communities.


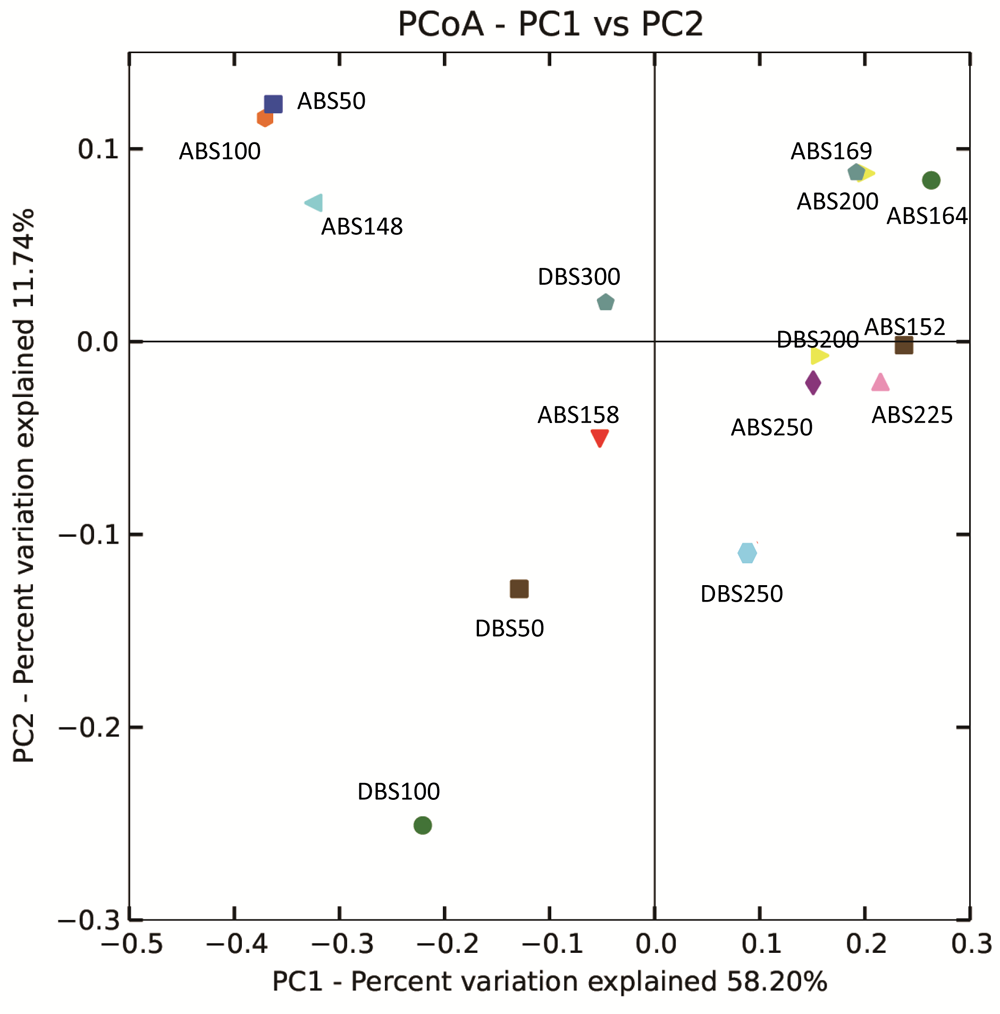


The relative abundances of OTUs defined at a 3% dissimilarity were used to calculate weighted UniFrac distance, followed by a PCoA analysis. PC1 and PC2 of the PCoA result were selected to plot the individual samples. The samples IDs were described in Table 2. This PCoA analysis did not take the samples, ABS175 and DBS150, into consideration, due to their low sequencing depth.
