# Supplementary material for: Zonation of Microbial Communities by a Hydrothermal Mound in the Atlantis II Deep (the Red Sea)
Source: PLoS One. 2015 Oct 20;10(10):e0140766. doi: 10.1371/journal.pone.0140766 (PMC4613831; doi:10.1371/journal.pone.0140766)
Supplement: S2 Fig — The relative abundance of the DHVEG1 and Thaumarchaeota group C3 was shown as their percentage in the individual communities. The sample IDs were referred to Table 2. (DOCX) [file pone.0140766.s002.docx]

S2 Fig. Relative abundance of DHVEG1 and group C3


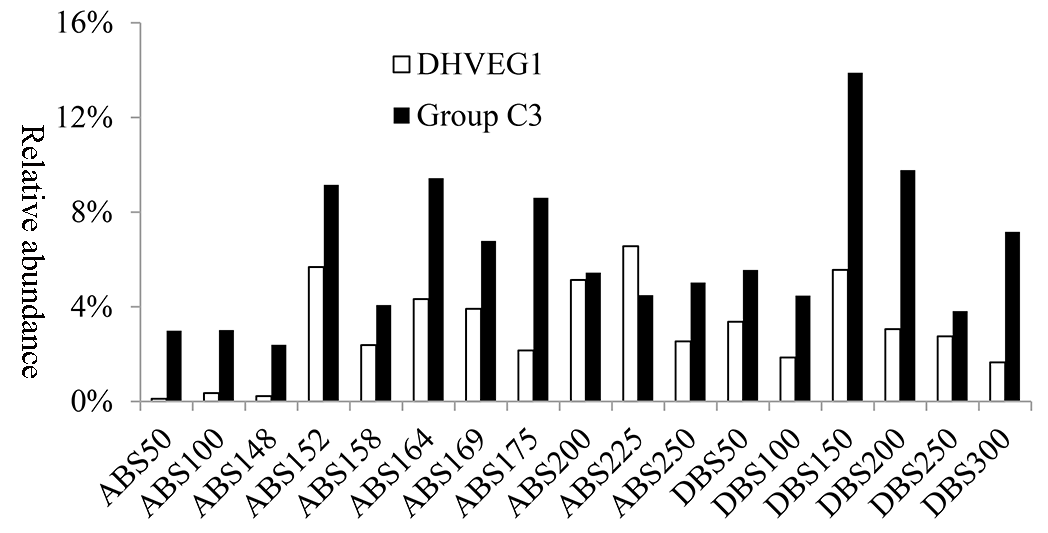


The relative abundance of the DHVEG1 and Thaumarchaeota group C3 was shown as their percentage in the individual communities. The sample IDs were referred to Table 2.
